# Percutaneous Device Closure of Congenital Isolated Ventricular Septal Defects: A Single-Center Retrospective Database Study Amongst 412 Cases

**DOI:** 10.1007/s00246-020-02315-0

**Published:** 2020-02-13

**Authors:** Varsha Walavalkar, Shreesha Maiya, Suresh Pujar, Prakash Ramachandra, Satheesh Siddaiah, Bart Spronck, Ward Y. Vanagt, Tammo Delhaas

**Affiliations:** 1 Department of Pediatric Cardiology and Grown-Up Congenital Heart Diseases, Narayana Institute of Cardiac Sciences, NH Health City, 258/A, Bommasandra Industrial Area, Anekal Taluk, Bangalore, Karnataka 560099 India; 2 Department of Biomedical Engineering, School of Engineering & Applied Science, Yale University, New Haven, CT USA; 3 Pediatric Cardiology, University Medical Center, Groningen, The Netherlands; 4 Cardiovascular Research Institute Maastricht CARIM, Maastricht University, Maastricht, The Netherlands

**Keywords:** Congenital ventricular septal defect, Percutaneous device closure, Amplatzer duct occluder, LifeTech ductal device

## Abstract

To identify suitable cases and reduce failure/complication rates for percutaneous ventricular septal defect (VSD) closure, we aimed to (1) study causes of device failure and (2) compare outcomes with different VSD types and devices in a high-volume single center with limited resources. Retrospective data of 412 elective percutaneous VSD closure of isolated congenital VSDs between 2003 and 2017 were analyzed. Out of 412, 363 were successfully implanted, in 30 device implantation failed, and in 19 the procedure was abandoned. Outcome was assessed using echocardiography, electrocardiography, and catheterization data (before procedure, immediately after and during follow-up). Logistic regression analyses were performed to assess effects of age, VSD type, and device type and size on procedural outcome. Median [interquartile range] age and body surface area were 6.6 [4.1–10.9] years and 0.7 [0.5–1.0] m^2^, respectively. Device failure was not associated with age (*p* = 0.08), type of VSD (*p* = 0.5), device type (*p* = 0.2), or device size (*p* = 0.1). Device failure occurred in 7.6% of patients. As device type is not related to failure rate and device failure and complication risk was not associated with age, it is justifiable to use financially beneficial ductal devices in VSD position and to consider closure of VSD with device in clinically indicated children.

## Introduction

Ventricular Septal Defect (VSD) is the most common congenital heart disease accounting for about 20% of all congenital heart defects in isolation [1]. Though VSDs can occur in any part of the interventricular septum, the majority are perimembranous. The remainder are muscular (inlet, posterior, mid, outlet or anterior) in location, whereas less than10% are supracristal [2]. In very rare cases, VSDs can be acquired such as ventricular septal rupture following myocardial infarction.

VSDs are commonly closed by open-heart surgery under cardiopulmonary bypass. Though surgical closure of a VSD is performed by experienced cardiac surgeons in dedicated cardiac hospitals, there remains a risk of bypass-related myocardial injury, complete AtrioVentricular Block (cAVB), blood transfusion [3], longer intensive care unit (ICU)/hospital stay and higher chances of developing infections postoperatively as compared to nonsurgical interventions.

Transcatheter closure of VSD with a device is an alternative to surgery in selected anatomical variants of VSDs. The major advantage with the recent advances in percutaneous device closure is that it reduces the hospital stay significantly [3].

To analyze the outcome of VSD device closure, it’s imperative to know success rate, causes of device failure as well as complications that occurred during the procedure and in the post-procedural period. To that end we assessed the outcome of percutaneous device closure in various anatomical variants of VSDs while using different devices including alternative (nitinol wire) ductal devices such as Amplatzer Duct Occluder 1 (ADO1), Amplatzer Duct Occluder 2 (ADO2) (AGA medical corporation, Minnesota, USA) and LifeTech ductal device (LifeTech scientific, Shenzhen, China) in VSD position.

## Methods

Retrospective data were collected of all patients who were taken for percutaneous device closure of congenital VSDs between January 2003 and December 2017 from hospital database. Pre-procedure electrocardiogram (ECG), echocardiography, chest radiograph and blood tests (Prothrombin time, International Normalized Ratio, Activated Partial Thromboplastin Time and platelet count to rule out any bleeding disorders); during procedure hemodynamic catheterization data and fluoroscopy time; immediate post-procedural and follow-up ECG, echocardiography and any complication/re-admission.

ECG and echocardiography was performed before and after device procedure (most by interventional cardiologists themselves) to assess the rhythm, exact location of the VSD, associated lesions, pulmonary arterial pressures, residual shunt, new onset of aortic and tricuspid regurgitations and pericardial effusion. Details of any complication during the course of hospitalization and subsequent treatments were noted.

Procedure: Access was via femoral artery and femoral or right internal jugular vein. Majority of VSDs were crossed from LV to RV using guide catheter and Terumo wire (Ashitaka factory of Terumo corporation, Fujinomiya city, Japan). The wire was advanced into the right/left pulmonary artery (RPA/LPA) and then snared out from the venous end using goose neck snare, forming an arteriovenous loop (AV loop). The delivery sheath/ guiding catheter was advanced over the wire covering the guide wire (Kissing technique) from the venous side. The tip of the delivery sheath was then positioned into the left ventricular (LV) apex (no special manipulation used). Thereafter, the device was deployed through the delivery sheath under transthoracic echocardiography and fluoroscopic guidance. In some cases, the VSD was crossed from the LV side, using a right coronary artery (RCA) guiding catheter which tip was placed in the RV. In these cases, the device was positioned from retrograde approach (no AV loop). Post-deployment LV and aortic angiograms were performed to assess any residual shunt.

Cases were divided into three categories: 1. Successful—where the device was successfully implanted into the heart; 2. Failed—where the device was selected and inserted into the heart and was found unsuitable while deploying the device; and 3. Abandoned—where the defect was found unsuitable for device closure after angiographic imaging, prior to opening the device from the company package.

## Statistical analysis

Statistical analyses were performed using IBM SPSS Statistics for Windows (IBM Corp. Released 2017, Version 25.0, Armonk, NY). Continuous variables were expressed as median with interquartile range. Categorical data were expressed as frequency with percentage.

Associations of outcome, device type, or VSD type with other predictors were assessed using (binomial or multinomial) logistic regression analyses. For potential continuous predictors, linearity was assessed by verifying that an additional quadratic term did not significantly improve modeling as quantified by a likelihood ratio (LR) test.

Regression models including VSD type were based on data for the four most common VSD types (1, 2, 4, and 5; Table 2) as inclusion of all types would lead to over-determination of logistic regression models. For the same reason, regression models including device type were based on data for device types 1, 2, 3, 4, and 9; Table 3. *P* values less than 0.05 were considered statistically significant.

## Results

Between January 2003 and December 2017, data on 433 percutaneous VSD device closure were retrieved by retrospective collection from hospital medical records. After exclusion of 21 cases (incomplete data, non-congenital acquired VSD), we analyzed 412 cases. Patient characteristics showing all three categories (successful, failed and abandoned procedure) are listed in Table 1.

**Table 1:** Patient characteristics stratified to VSD device outcome

Variable (unit)	VSD device outcome		
	Successful (*n* = 363)	Failed (*n* = 30)	Abandoned (*n* = 19)
Age (years)	6.6 (4.1–10.9)	5.6 (3.7–8.5)	5.5 (3.2–9.1)
BSA (m^2^)	0.7 (0.5–1.0)	0.6 (0.6–0.8)	0.7 (0.5–1.1)
SPO_2_ (%)	100 (98–100)	100 (98–100)	100 (98–100)
Heart rate (bpm)	100 (100–115)	100 (98–110)	110 (100–120)
VSD size (mm)	4 (3–4)	4 (4–5)	4 (4–5)
QP/QS	2.0 (1.6–2.3)	2.0 (1.7–2.3)	1.8 (1.7–2.0)
Fluoroscopy time (min)	14.1 (9.2–21.2)	18.9 (15.0–29.2)	17.3 (8.2–28.7)
Follow-up (days)	246 (71–764)		

Values denote median (interquartile range)

*BSA* body surface area, *QP/QS* pulmonary/systemic flow ratio, *SPO*_*2*_ peripheral oxygen saturation, *VSD* ventricular septal defect

15 different types of devices were found in the database with 10 different anatomical variants of VSDs (Tables 2 and 3). We studied the association of majorly used VSD devices (1, 2, 3, 4 and 9) and higher numbered VSDs (1, 2, 4, and 5) with device failure and association between Chinese versus American and muscular versus ductal devices in VSD position using logistic regression analysis.

**Table 2:** Variants of VSD types

VSD type	Total	Successful	Failed	Abandoned
Perimembranous (1)	136 (33%)	120 (88%)	6 (4.4%)	10 (7.4%)
Posterior upper muscular (2)	156 (38%)	137 (88%)	14 (9.0%)	5 (3.2%)
Perimembranous upper muscular (3)	3 (0.7%)	2 (67%)	1 (33%)	0 (0.0%)
Upper muscular (4)	69 (16%)	62 (89%)	5 (7.2%)	2 (2.8%)
Mid muscular (5)	26 (6%)	23 (88%)	2 (7.6%)	1 (3.8%)
Lower muscular (6)	5 (1.2%)	4 (80%)	1 (20%)	0 (0.0%)
Anterior upper muscular (7)	7 (1.7%)	6 (85%)	1 (14%)	0 (0.0%)
Posterior mid muscular (8)	8 (2%)	8 (100%)	0 (0.0%)	0 (0.0%)
Anterior muscular (9)	1(0.25%)	1 (100%)	0 (0.0%)	0 (0.0%)
SubAortic (10)	1 (0.25%)	0 (0.0%)	0 (0.0%)	1 (100%)

Values in first (total) column denote absolute frequency and percentage of each variant of VSD out of the total VSDs. The other columns denote the frequency and percentage of successful, failed and abandoned cases for each VSD variant

**Table 3:** Variants of VSD devices and success/failure rate

Device type	Total	Successful	Failed
Amplatzer ductal occluder-1 (1)	49 (12.5%)	43 (88%)	6 (12%)
Amplatzer ductal occluder-2 (2)	41 (10.4%)	35 (85%)	6 (15%)
LifeTech ventricular septal defect (3)	37 (9%)	32 (86%)	5 (13%)
LifeTech patent ductus arteriosus (4)	150 (38%)	142 (94%)	8 (5%)
LifeTech perimembranous (5)	2 (0.5%)	2 (100%)	0 (0.0%)
Amplatzer-muscular (6)	18 (4.5%)	18 (100%)	0 (0.0%)
Amender patent ductus arteriosus (7)	11 (2.8%)	11 (100%)	0 (0.0%)
Amender-ventricular septal defect (8)	7 (2%)	7 (100%)	0 (0.0%)
CardioFix ventricular septal defect (9)	38 (10%)	35 (92%)	3 (8%)
CardioFix-patent ductus arteriosus (10)	16 (4%)	16 (100%)	0 (0.0%)
CardioFix perimembranous (11)	4 (1%)	4 (100%)	0 (0.0%)
Blockade muscular (12)	13 (3.3%)	13 (100%)	0 (0.0%)
Blockade perimembranous (13)	4 (1%)	2 (50%)	2 (50%)
Cocoon (14)	2 (0.5%)	2 (100%)	0 (0.0%)
C-cure (15)	1 (0.5%)	1 (100%)	0 (0.0%)

Values in first (total) column denote absolute frequency and percentage of each variant of VSD device out of the total devices. The other columns denote the frequency and percentage of rate of successful and failed cases for each VSD device variant

**Fig. 1:**
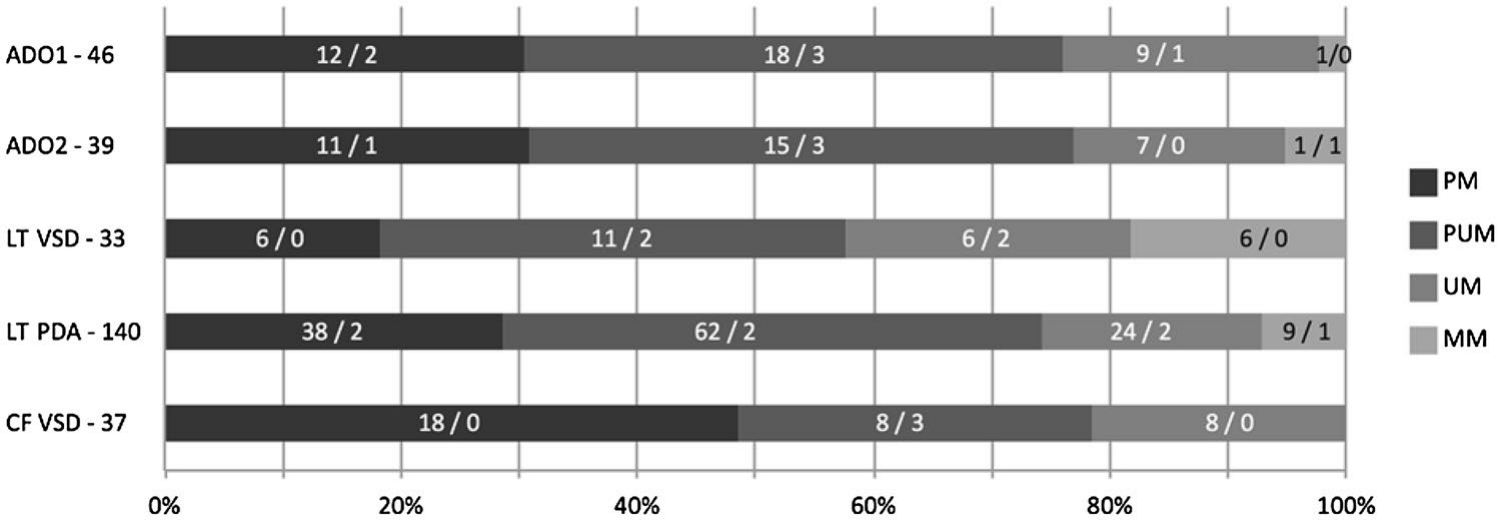
Distribution of cumulative frequencies of major VSD and device types between successful/failed. Device type and total number of its implantations are depicted next to the Y-axis on the left. The width of each horizontal block depicts the cumulative frequencies of the device used per different VSD type, with the absolute number of successful/failed cases depicted within the horizontal block. *ADO1/2* Amplatzer ductal occluder ½, *CF* CardioFix, *LT* LifeTech, *MM* mid muscular, *PM* perimembranous, *PDA* patent ductus arteriosus, *PUM* posterior upper muscular, *UM* upper muscular, *VSD* ventricular septal defect

**Fig. 2:**
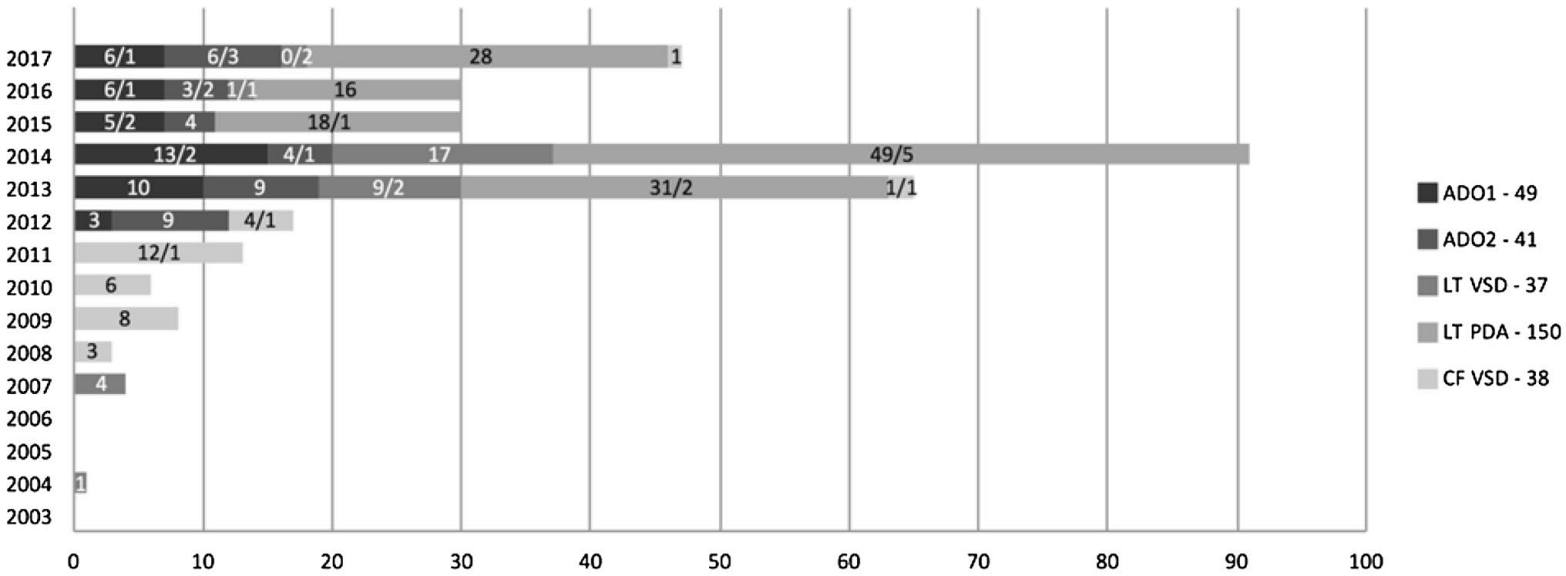
Distribution of five major VSD device types between successful/failed cases over years. Device type and total number of the corresponding device are depicted next to the Y-axis on the right. The width of each horizontal block in different shades depicts the cumulative frequency of the type of device used each year over 15 years, with the absolute number of successful/failed cases within the horizontal block. *ADO1/2* Amplatzer ductal occluder 1 or 2, *CF* CardioFix, *LT* LifeTech, *PDA* patent ductus arteriosus, *VSD* ventricular septal defect

The distribution of major types of VSD and devices between successful and failed cases is shown in Fig. 1 and the distribution of 5 majorly used devices between successful and failed cases over 15 years is shown in Fig. 2.

No significant association was found between probability of device failure and subject age (*p* = 0.085), weight (*p* = 0.310), BSA (*p* = 0.382), or sex (*p* = 0.373; binomial logistic regression, LR tests).

Probability of device failure was also not significantly associated with VSD type (*p* = 0.536, binomial logistic regression, LR test) or with device type (*p* = 0.228, binomial logistic regression, LR test). There was also no difference in failure rate between muscular and ductal devices (p = 0.332, binomial logistic regression, LR test).

Devices were further divided into two categories based on the manufacturing country (United states and China). Probability of device failure was not significantly associated with manufacturing country (United States vs. China; *p* = 0.090, binomial logistic regression, LR test).

Out of 15 different type of VSD devices, LifeTech Patent Ductus Arteriosus (PDA) device was used most frequently (38%) with interestingly the lowest failure rate (5%); however, the difference was not statistically significant from other devices (*p* = 0.228) which could be due to lack of statistical power, as some device types were used in very small numbers.

There was no significant association between device size and probability of device failure (*p* = 0.168; binomial logistic regression, LR test), probability of aortic regurgitation (AR) (*p* = 0.087; binomial logistic regression, LR test) and tricuspid regurgitation (TR) (*p* = 0.773; multinomial logistic regression, LR test).

Probability of success vs. failure significantly decreased with fluoroscopy time (*p* = 0.005 (LR test; *χ*^2^(1) = 7.82), *β* = − 0.048 (SE 0.017), binomial logistic regression with fluoroscopy time as a continuous predictor). Specifically, each additional minute of fluoroscopy time corresponds to a 4.7% reduction in the odds of a successful outcome. This indicates that failed device closures had longer radiation time which we attribute to technical difficulty.

The prevalence of new onset of AR (grade-1) was 15% immediate after device closure and reduced to 5% at median follow-up of 246 days (range 71–764). In the immediate post-device period, grade-1 TR was present in 48%, but this number reduced to 22% at follow-up. Mild left ventricle to right atrium (LV-RA) jet was present in 3% of the cases in immediate post-procedure period, and reduced to less than 1% at follow-up. 3% of patients had tiny residual VSD at follow-up and 3% of patients had minor vascular complications in the immediate post-device period which did not require any intervention (Table 4).

**Table 4:** Outcome variables pre-device, immediate post-device and follow-up

Outcome variable	Time interval		
	Pre-device (*n* = 363)	Immediate post-device within 24 h (*n* = 363)	Follow-up (median 246 (71–764) days) (*n* = 135)
Aortic regurgitation (grade-1)	2 (0.5%)	53 (14%)	7 (5%)
Tricuspid regurgitation (grade-1)	20 (5.5%)	173 (47%)	30 (22%)
LV-RA jet (Mild)	89 (24%)	10 (2.8%)	1 (0.7%)
Residual VSD (Tiny)	–	34 (9.5%)	4 (3%)
Vascular complications	–	12 (3%)	–

Complications which needed intervention	Time period post-device	Cause	Frequency (%)
Aortic valve repair (surgical)	2 months	Torn NCC	1 (0.28%)
Tricuspid valve repair (surgical)	5 months	Chordal rupture	1 (0.28%)
cAVB, permanent pacemaker implantation	1 and half year	cAVB	1 (0.28%)
Embolization	Next day	Embolized in distal LPA	1 (0.28%)
Observation 1st degree AV Block with LBBB	6 weeks	EP study didn’t warrant permanent pacemaker implantation	1 (0.28%)
Death	5 days	Intracranial bleeding	1 (0.28%)

Values denote frequency and percentage of each complication

*cAVB* complete atrioventricular block, *EP* electrophysiological, *LBBB* left bundle branch block, *LPA* left pulmonary artery, *NCC* non-coronary cusp

Two patients had to undergo open-heart surgery due to major complications (tear in the aortic valve cusp and tricuspid chordal rupture); one patient had device embolization 5 days after the device procedure and one patient died due to intracranial bleeding (Table 4). The complications that required intervention are explained in detail in the discussion section.

There was no major conduction or rhythm abnormality in the early post-procedure period. One patient, however, was re-admitted after 6 weeks with intermittent left bundle branch block (LBBB) with 1st degree atrioventricular (AV block) (0.28%). Because the patient’s 24-h Holter monitoring did not warrant permanent pacemaker implantation, the patient was discharged and was advised follow-up. Out of 363 successful percutaneous VSD device closure cases, one case developed cAVB requiring permanent pacemaker implantation (0.28%) at follow-up of 1 year and 5 months.

## Discussion

The present study reports the evaluation and comparison of different anatomical type of VSDs, various devices and the complications. Though various studies have shown good results previously [4–6], pre-procedural prediction of complications is difficult. We evaluated the cases from the pre-procedure period to follow-up for overall outcome and feasibility of using alternative devices (like PDA device) in VSD position.

According to our observation there was no significant association between device failure and patient’s age, weight, body surface area, type of VSD or the device used. Of note, device selection was decided depending on VSD morphology rather than age at procedure with, preferentially, ductal devices for VSDs in membranous/upper muscular location, muscular VSD devices for muscular VSDs, and ADO2 with their softer profile for VSDs close to aortic valve. VSD device closure procedure done at an experienced center can be successfully performed in carefully selected cases (preferably by interventional cardiologists themselves) with suitable morphology (VSD location and its relation to conduction tissue, and aortic/tricuspid valve).

Historically, children under age of 3 years undergoing device closure for VSDs located in the membranous region are considered to be at risk for developing cAVB [5, 7–9]. cAVB becomes important especially for VSDs located in membranous and inlet septum (due to their proximity to the AV node), whether the approach is surgical or with a device [6, 9].The incidence of heart block in surgically operated isolated VSDs requiring permanent pacemaker implantation is 28 (2.8%) out of 1000 cases in our high-volume center (unpublished data). In this series of 412 percutaneous VSD device closures, only one case developed cAVB. This was a 3-year-old child with a 4 mm posterior upper muscular VSD on echocardiography with mild LV-RA jet who had successful VSD closure with an 8/6 mm LifeTech PDA device. After device procedure there was no residual shunt, no LV-RA jet and no rhythm abnormality at the time of discharge. However, this patient presented to the hospital after 1 year 5 months post-procedure with cAVB (0.28%) for which epicardial VVIR pacemaker was implanted. This delayed complication of device closure, possibly due to fibrous tissue formation around the device [4], indicates that regular follow-up is needed because one cannot completely exclude the possibility of delayed cAVB despite an uneventful immediate post-device period. Regarding type of device selection in relation to the age, was decided depending on the morphology of VSD rather than the age at procedure.

None of the five most commonly used devices had statistically significant outcome predictors related to four majorly closed types of VSDs. This indicates that, when carefully chosen, neither the type of device nor various anatomical locations of VSDs influence the outcome. Interestingly the median VSD size in our population was 4 mm as VSDs in membranous/upper muscular location are usually restricted by tricuspid valve tissue or septal aneurysm on RV side, which makes them conical in shape. Hence, ductal devices (mainly ADO1 type) can be safely used in VSD position, particularly in perimembranous location, as it has only single disc on LV side and no disc on right ventricular (RV) end. We believe this is the major reason for the low incidence of cAVB and tricuspid valve issues. On the other hand muscular VSD devices are better suited for muscular VSDs. ADO2 are softer profile devices which can be considered for VSDs close to aortic valve. Larger VSDs are closed with large sized devices provided they are not close to aortic valve or conduction tissue.

It is important to look for new onset of valve regurgitation after the procedure. Because 33% of the cases from our database had perimembranous VSD, which is in close proximity to aortic valve on LV side and tricuspid valve on RV side, careful assessment of AR and TR becomes important [4]. In our 363 successful cases, one patient had severe TR (4 mm muscular VSD device) at follow-up of 5 months; echocardiography showed torn chordae of tricuspid valve for which surgical tricuspid valve repair was done. Another patient developed severe AR at follow-up of 2 months requiring aortic valve repair. At surgery it was noted that the device was away from the aortic valve, hence it was left in situ and only aortic valve repair was done. We believe that the AR was possible secondary to aortic valve damage while performing AV loop or pushing the delivery sheath across the valve (iatrogenic). Rest of the cases who had documented grade I AR (similar to trace AR-more physiological than pathological AR) in post-procedure period could be partly due to catheter manipulation and inter-observer variation in assessment of presence of AR. As being the main center where the procedure was done we have not had re-do for aortic valve except for one (above mentioned) case after device closure.

Of all the successful cases, 24% had LV-RA jet associated with VSD (mainly perimembranous/posterior upper muscular) which reduced to 2.8% immediately after the procedure and to 0.7% at follow-up. This shows that, due to endothelialization and fibrosis, the device can eventually also cover the LV-RA jets associated with a VSD, especially in the region of pars atrioventricularis.

In the immediate post-procedure period, 34 (9%) patients had tiny residual leak out of which 1 was hemodynamically significant (0.28%) with large L-R shunt. Interestingly we observed that at median follow-up of 246 (71–764) days small residual leaks reduced to 4 (3%) cases. This could be due to the endothelialization of the device that can close the tiny residual leaks usually seen immediately after the procedure.

Large device size and longer fluoroscopy time are of concern for developing post-procedure arrhythmias [6]. In our study fluoroscopy time was longer for failed device and abandoned procedure categories; this could be due to technical difficulty in crossing the VSD.

There was no association of device size and device failure or rhythm abnormality. One patient developed transient atrioventricular (AV) block with atrial fibrillation during closure with ADO1 device of posterior upper muscular VSD. Because the patient reverted to sinus rhythm on table, the device was successfully deployed. Though this patient was re-admitted with 1st degree AV block with LBBB, no further intervention was done as 24 h holter monitoring was normal. Though LBBB is a rare complication seen after device closure [4, 7, 10], it’s important to advice for early follow-up and review ECG (less than 4 weeks) for patients who had rhythm abnormality during the procedure.

Technical difficulty in crossing the defect was found in VSDs located in lower muscular septum and in VSDs covered with large septal aneurysms with multiple sieve-like openings.

Hemolysis is another complication that is known to happen soon after device closure in 1% of cases [4, 7]. Hemolysis occurs more commonly in cases with significant residual shunts after device closure, due to mechanical injury to red blood cells. Hemolysis can be minimal and resolve on its own. However, when severe it may warrant urgent surgical removal of the device [11]. In our observation from the database there were no cases with hemolysis.

In our study the only patient with significant residual VSD post-procedure was unresponsive within 12 h after procedure due to intracranial bleeding and subsequently died. Patient’s pre-procedure coagulation status was normal. Our postulation is bleeding might have been secondary to (a) intravenous heparin as per protocol (100 UNIT/Kg body weight), (b) intracranial pathology such as aneurysm in the circle of Willis etc. and (c) acute hemodynamic changes that occur during induction of anesthesia (e.g., ketamine used in this patient could have increased intracranial pressure), or (d) more likely to be a combination of above factors.

## Limitations

The main limitation is that the study is retrospective (complete availability of the data we can access from records is not in our control), we lack the long-term follow-up, which is very important to assess possible late complications and it is a single-centered study.

## Conclusion

In our observation device failure was neither associated with the age at procedure nor with the type of VSD or device used. Considering the low incidence of major complications, it does not seem necessary to postpone percutaneous VSD closure when indicated, even in smaller children. As ductal devices were used in VSD closure in majority of cases, it is justifiable to use financially beneficial ductal devices in VSD position. However, long-term follow-up study to rule out late complications is indicated.
